# Differentiated Type II Pneumocytes Can Be Reprogrammed by Ectopic Sox2 Expression

**DOI:** 10.1371/journal.pone.0107248

**Published:** 2014-09-11

**Authors:** Joshua Kapere Ochieng, Kim Schilders, Heleen Kool, Marjon Buscop-van Kempen, Anne Boerema-De Munck, Frank Grosveld, Rene Wijnen, Dick Tibboel, Robbert J. Rottier

**Affiliations:** 1 Department of Pediatric Surgery of the Erasmus MC-Sophia Children's Hospital, Rotterdam, the Netherlands; 2 Department of Cell Biology of the Erasmus MC, Rotterdam, the Netherlands; Helmholtz Zentrum München/Ludwig-Maximilians-University Munich, Germany

## Abstract

The adult lung contains several distinct stem cells, although their properties and full potential are still being sorted out. We previously showed that ectopic Sox2 expression in the developing lung manipulated the fate of differentiating cells. Here, we addressed the question whether fully differentiated cells could be redirected towards another cell type. Therefore, we used transgenic mice to express an inducible Sox2 construct in type II pneumocytes, which are situated in the distal, respiratory areas of the lung. Within three days after the induction of the transgene, the type II cells start to proliferate and form clusters of cuboidal cells. Prolonged Sox2 expression resulted in the reversal of the type II cell towards a more embryonic, precursor-like cell, being positive for the stem cell markers Sca1 and Ssea1. Moreover, the cells started to co-express Spc and Cc10, characteristics of bronchioalveolar stem cells. We demonstrated that Sox2 directly regulates the expression of Sca1. Subsequently, these cells expressed Trp63, a marker for basal cells of the trachea. So, we show that the expression of one transcription factor in fully differentiated, distal lung cells changes their fate towards proximal cells through intermediate cell types. This may have implications for regenerative medicine, and repair of diseased and damaged lungs.

## Introduction

The mammalian lung is a complex organ with a large and highly vascularized epithelial surface area. The airway epithelium is lined with a diversity of cell types that vary in abundance along the proximal-distal axis. The conducting airways have a pseudostratified epithelium to facilitate mucociliary transport, which gradually transforms into a simple columnar and cuboidal epithelium. Finally, the respiratory part of the lungs consists of squamous epithelium for efficient gas exchange. Cellular homeostasis is important for the maintenance of the lung, and in mature lungs, cell turnover and proliferation is low [1]. However, after bronchiolar injury, either infections or mechanical insults such as artificial ventilation to the lung, the respiratory epithelium extensively proliferate to regenerate and repair the injured lung, indicating the presence of lung progenitor cells [2], [3].

In general, lung stem/progenitor cells should have the capacity to self-renew and differentiate into specialized cell lineages. In mouse, endogenous adult progenitor/stem cells function to repopulate the damaged lung epithelium [4]–[6]. Several distinct populations of stem/progenitor cells have been described to be present in the conducting and respiratory epithelium [2], [6]–[10]. Lineage tracing studies in mice have shown that the proximal airway basal cells act as stem cells, giving rise to Clara and ciliated cells during lung injury [11], [12]. On the other hand, recent data suggest that Clara cells may differentiate into Trp63 positive basal cells in damaged lung parenchyma and into alveolar type II cells upon bleomycin treatment or influenza infection [2], [13]. Other putative proximal stem cells include a subpopulation of toxin-resistant Clara cells that function as bronchiolar stem cells located within two discrete cell niches: the neuroepithelial body (NEB) and the bronchoalveolar duct junction (BADJ) [11], [14], [15]. Moreover, several studies have shown the differentiation of type II cells into type I cells [2], [16]. Thus, intrinsic cell populations exist in the lung that may be triggered to differentiate into distinct cell types.

Sox2 is among other transcription factors essential for lung development and maturation [17]–[19]. Sox2 is a member of the highly conserved HMG box family of transcription factors and required early in embryonic development to maintain pluripotency and self-renewal in embryonic stem (ES) cells. In mice, Sox2 is required for normal morphogenesis and homeostasis of diverse tissues, including neural stem cells; retinal stem cells taste buds; hair sensory follicles in the ear; and epithelia of trachea, lung, and esophagus [17], [20]–[22]. Sox2 is one of the original factors together with Oct4, Klf4, and c-Myc required for the reprogramming of somatic cells [23]. In the embryonic lung, Sox2 is expressed in the developing respiratory epithelium [18], whereas in adult lungs, expression of Sox2 is restricted in epithelial cells, in the adult trachea, airway/bronchiolar epithelium and the conducting airways. [17], [24], [25]. Sox2 is completely absent from the respiratory airways, where another member of the Sry-box family, Sox9, is expressed. Thus, Sox2 and Sox9 show a reciprocal expression pattern in the lung.

Many reports have described variations of the original cocktail of factors to generate multipotent iPS cells in vitro (reviewed in [26], [27]). Lineage conversion or trans differentiation have recently been reported in vitro and in vivo (review [28]–[31]. Mouse and human fibroblasts and other types of cells have been trans-differentiated directly into post-mitotic neurons with combinations of transcription factors [32]–[37]. It was recently reported that the combination of three or more factors can reprogram mouse fibroblasts into induced neural stem cells (iNSCs) with self-renewing ability [38]–[41].

Recently, we showed that ectopic expression of Sox2 during lung development induced the differentiation of embryonic epithelial cells into basal and neuroendocrine cells [18]. Since subsets of epithelial cells in the developing lung may still be multipotent, we wondered whether ectopic expression of Sox2 could change the faith of fully differentiated alveolar type II cells in vivo. Therefore, we ectopically expressed Sox2 in alveolar type II cells using a tet-inducible, bi-transgenic approach. We show that conditional expression of Sox2 in the alveolar epithelium results in emphysematous lungs concomitant with the emergence of aberrant structures containing cuboidal cells in the periphery of the lungs. Moreover, Sox2 was found to induce progenitor-like cells which become proliferative and differentiate into cuboidal and basal-like cells, implying that fully differentiated type II cells can be reprogrammed with a single transcription factor to develop into cells expressing proximal markers.

## Materials and Methods

### Mouse breeding and genotyping

Mice were kept under pathogen-free conditions and all experiments described in this study were performed according to the guidelines and with the written approval of the local ethics committee, “Dier Ethische Commissie” (Animal Ethics Commission), permit number EMC 2206. Generation of the Sox2 transgenic mouse line has been described before [18]. Lung-specific expression of the myc-Sox2 transgene was obtained by breeding the myc-Sox2 line with the SPC-rtTA transgenic mice (generous gift of Jeffrey Whitsett, Cincinnati), subsequently referred to as iSox2^SPC-rtTA^. Doxycycline was administered in the drinking water (2 mg/ml doxycycline, 5% sucrose), and lungs were harvested after 1, 3, 6, 9, or 28 days. Transgenic mice were genotyped by PCR of tail-tip DNA using transgene specific primers as previously described. Each experiment was executed on at least three independent lungs of iSox2^SPC-rtTA^, single transgenic and wild type pups.

### Immunohistochemistry and Immunofluorescence

Lungs of adult mice were inflated with 4% paraformaldehyde/phosphate-buffered saline (PBS), subsequently fixed by immersion overnight at 4°C, and processed according to standard protocols for paraffin embedding. Immunohistochemistry (IHC) was performed as previously described using primary antibodies for goat-anti Sox2 (1∶500 immune system), mouse anti myc (1∶1000, Roche) rabbit anti-phospho-histone H3 (1∶1000; Millipore), rabbit anti-CCSP (1∶1000, 7-Hills), Mouse anti Ssea (1∶200, Millipore), rabbit anti-cyclin D1 (1∶500; Abcam), rat anti-Sca-1 (1∶500; Abcam), rabbit anti-proSP-C (1∶1000; Seven Hills), Mouse anti-Trp63 (1∶200; Santa cruz), and Goat anti-CCSP (1∶1,000; Seven Hills) [42]. Briefly, sections (5 µm) were deparaffinised, rehydrated and microwave treated for antigen retrieval in 10 mM citric acid buffer, pH 6.0. Slides were incubated with primary antibodies diluted in PBS/0,5% Triton/0.5% BSA overnight at 4°C, followed by incubation with the appropriate secondary antibody (1∶100; Vector Labs) and amplification with ABC reagent (Vectastain Elite ABC kit; Vector Labs). Antigen localization was detected with nickel-diaminobenzidine. Sections were counterstained with haematoxylin and coverslipped using Permount (Fisher Scientific). Quantification of IHC staining was performed by counting positive cells in five separate microscopic fields with a 40× objective of three independent experiments, after which a bar graph was prepared using PRISM graphpad. Data are represented as the average of the number of positive cells per field, including the standard error of the mean (SEM).

Immunofluorescence was performed as described above, but substituting the secondary antibodies with fluorophore labelled antibodies (Alexa Fluor-350, Alexa Fluor-488, and Alexa Fluor-594; Molecular Probes). Sections were mounted with Vectashield anti-fade reagent containing DAPI (Vector Labs). Brightfield and fluorescent images were obtained using a Zeiss Axioplan2 microscope equipped with AxioVision Software or a Leica SP5 confocal microscope.

### Isolation and culturing of alveolar type II cells (AVTII)

Alveolar epithelial cells were isolated from the lungs of Sox2^SPC-rtTA^ mice by Dispase (BD, Pharmingen) digestion as described previously with few modifications [43]. Lungs were exsanguinated by perfusing through the right ventricle with 4 ml PBS after opening the peritoneum, clipping the vena cava inferior and removing the ribcage. 1 ml Dispase (BD, Pharmingen) was instilled over a tracheal cannula into the lung, immediately a sterilized suture (Braun) was used to tighten a node around the cannulised trachea. Lungs were isolated, incubated for 45 minutes in 1 ml Dispase at room temperature and transferred to a culture dish containing 5 ml DMEM/F12 medium (Gibco) supplemented with 0.04 mg/ml DNase I (AppliChem), 3.6 mg/ml D-(+)-Glucose (AppliChem) and 1% Penicillin/Streptomycin (P/S). The small airways were gently removed and the obtained cell suspension was serially filtered through 100, 70 and 40 µm nylon meshes and centrifuged at 200 g for 10 minutes at 15°C. The supernatant was discarded and the cell pellet was resuspended in 500 µl DMEM/F12 (Gibco) medium supplemented with 3.6 mg/ml D-(+)-Glucose (AppliChem), 1% P/S and 2% FCS. AVTII cells were cultured in DMEM/F10 containing 10% FCS and 1% P/S in tissue culture cover slip immersed in 12 well plates (Corning, NY) previously coated with collagen (Inamed); cultures were maintained in a 5% CO_2_/air incubator.

### Plasmids, Cell culture and Luciferase Reporter Assays

A 500 bp minimal promoter fragment immediately upstream of the Sca1 transcriptional start site was PCR amplified from genomic DNA using the primers Forward 5′-TAAACGCGCACACGTTTCTC-3′ and Reverse 5′-GGCCAGCATCTGACCTCTTT-3′, and cloned into the pGL4 luciferase. Human embryonic kidney HEK 293T cells maintained under standard culture conditions were plated on 6-well plates (3.5×10^5^ cells per well). 24 hours after plating, the HEK-293Tcells were transiently transfected using Lipofectamine LTX (Invitrogen) with 2.5 µg of the following Firefly luciferase reporter plasmids (pGL4-Sca-1 1 µg of Renilla luciferase plasmid (transfection control), and 2.5 µg of empty vector (pcDNA3) or plasmids expressing full length Sox2 (WT-Sox2), or one of the mutant Sox2 (ΔTAD and ΔHMG)[44]. After 24 hr, cells were harvested and luciferase activity was measured using the Dual-Luciferase Reporter Assay system (Promega). The lysate was assayed for luciferase and Renilla activity using the GloMax 96 Microplate Luminometer with Dual Injectors (Promega, Madison, WI), according to the Dual-Luciferase Reporter Assay system kit protocol (Promega). The luciferase activity was calculated relative to the TK Renila. All reporter assays were performed in triplicate, and the bars in the figures denote the standard error of the mean (SEM).

### Chromatin Immunoprecipitation

Human lung adenocarcinoma epithelial A549 cells were cultured in hypoxic conditions and the ChIP assays were performed essentially as described previously using 6×10^7^ cells [45]–[47]. The immunoprecipitated DNA was analysed with specific primers in a Q-PCR assay to assess the enrichment of the promoter regions of the Amylase (Amy), Sca1 and Gli2 genes. The amount of immunoprecipitated DNA with SOX2 and control IgG was calculated based on threshold cycle [C(t)] using the ΔC(t) method and normalized to input samples. Results are expressed as fold enrichment of SOX2 immunoprecipitated samples relative to IgG controls, and represent the average of three replicates of two independent experiments including the SEM. PCR primers are listed in Table 1.

**Table 1:** Primers used for ChIP-qPCR.

Gene	orientation	sequence
SOX2	forward	5′ - CATGCACCGCTACGACG - 3′
	reverse	5′ - CGGACTTGACCACCGAAC - 3′
GLI2	forward	5′ - TAGAATTGCTCCTGCACTTC - 3′
	reverse	5′ - ATGTCGGATGACCCTTTCTC - 3′
AMY	forward	5′ - GGGAAAAGGCAGCATATTG - 3′
	reverse	5′ - CACGCTAAATTGCCTGTGAA - 3′
SCA1	forward	5′ - ATGCCTTTATAGCCCCTCT - 3′
	reverse	5′ - GTCATGAGCAGCAATCCACA - 3′

## Results

### Expression of Sox2 in alveolar type II cells results in morphological changes

**Figure 1:**
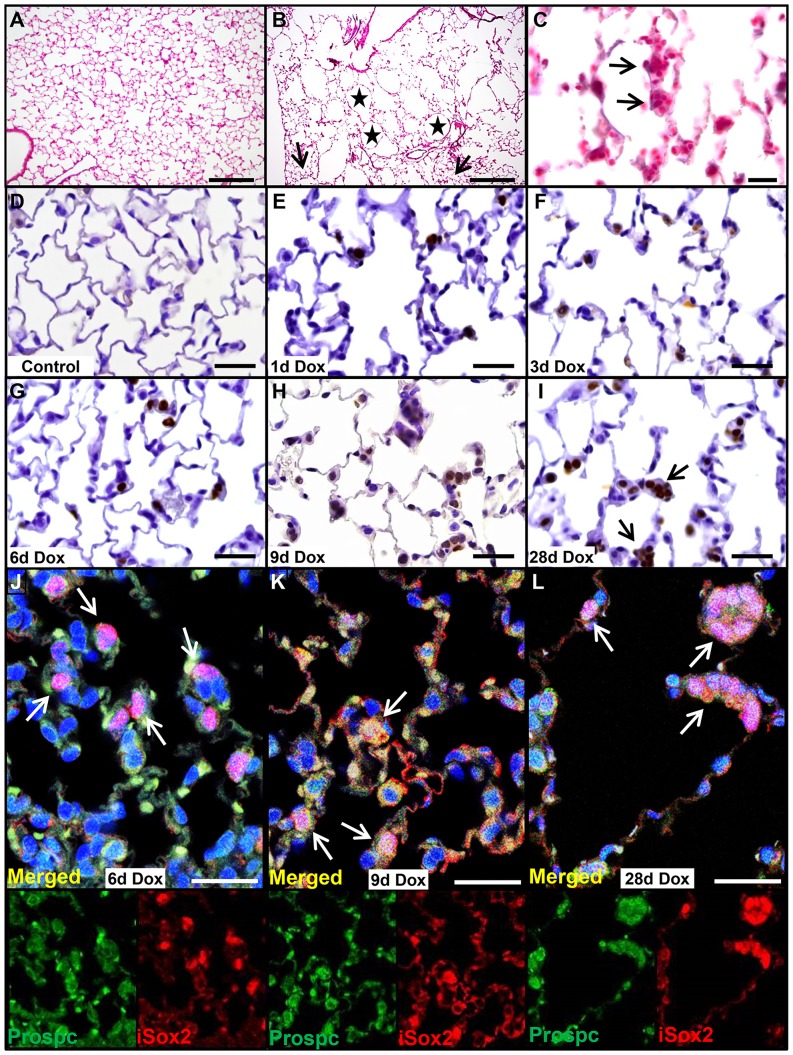
Ectopic Sox2 expression induces abnormal cell clusters. Representative HE staining of a control lung (A) demonstrating the normal lung architecture and of iSox2^SPC-rtTA^ lungs after 4 weeks doxycycline treatment (B) showing numerous, enlarged emphysematous structures (asterisks) with cuboidal cell clusters (C). Representative IHC staining for the myc-epitope in wild type control (D) and iSox2^SPC-rtTA^ lungs (E–I) after 1, 3, 6, 9 and 28 days of doxycycline treatment. Transgenic iSox2 positive myc staining is already evident after 1day of dox administration (E), which gradually increased in time (F–I). The positive cells are clearly forming cuboidal clusters (arrows in I). Dual immunofluorescence staining shows the colocalization of the transgenic Sox2 (iSox2, red) with the type II cell marker Prospc (green) after 6 days (J), 9 days (K) and 28 days (L) of doxycycline treatment. Scale bars 200 µm (A, B), 100 µm (C), 50 µm (D–I) and 25 µm (J–L).

**Figure 2:**
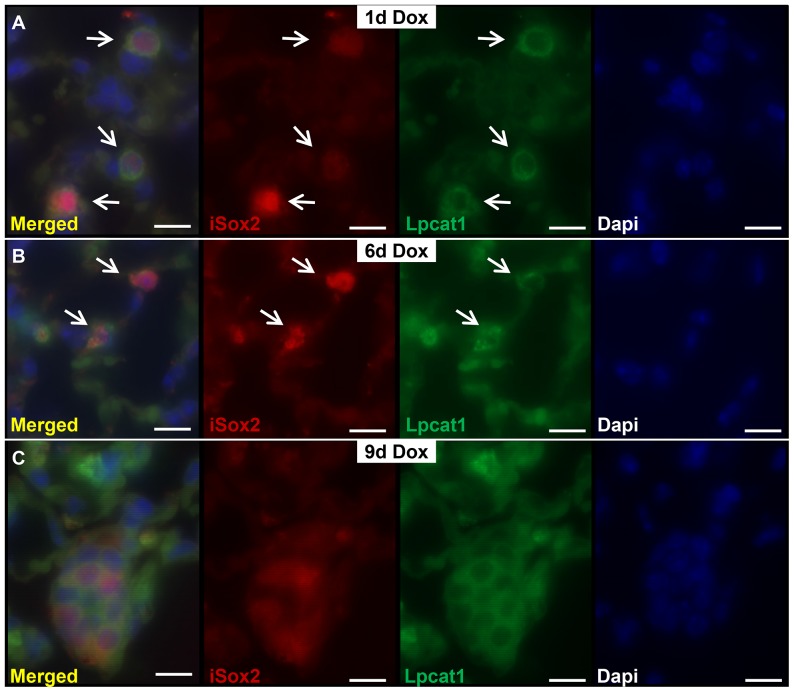
Sox2 induces a gradual loss of differentiated type II cells. Dual immunofluorescence staining of lungs treated with doxycycline for 1 (A), 6 (B) and 9 (C) days shows a gradual loss of colocalization of the type II cell differentiation marker Lpcat1 (green) and the transgenic Sox2 (iSox2, red), indicating that Sox2 induces cellular changes in the type II cells. Scale bar: 25 µm.

Ectopic expression of iSox2 in relative uncommitted, naïve epithelial cells during development resulted in their differentiation into proximal epithelial cells, primarily basal cells [18], [46]. We wondered whether induced expression of iSox2 in fully differentiated cells would affect the cells in an autonomous manner. Therefore, we expressed the iSox2 transgene in adult lungs using the SPC-rtTA transgene, which was reported to be expressed in a subset of type II cells in adult lungs [48]. The immediate effect of the expression of the transgene was evaluated after one, three, six, and nine days of doxycycline induction, as well as prolonged expression for four weeks. The mice did not show typical lung-related phenotypic abnormalities, such as breathing problems, and were indistinguishable from control mice. However, histological analysis of HE stained sections revealed significant structural abnormalities after treatment with doxycycline for four weeks in the lungs of the Sox2^SPC-rtTA^ mice, which resembled an emphysematous-like appearance (Figure 1B, stars). The air spaces in the lungs of iSox2^SPC-rtTA^ mice were significantly enlarged and accompanied by the destruction of the normal alveolar architecture compared to control mice (Figure 1A–C). The disrupted areas were interspersed within relative normal alveolar regions, indicating that the phenotype was not uniform throughout the lung of iSox2^SPC-rtTA^ mice. The normal appearance of lung architecture in some regions was an indication of incomplete penetrance of the transgene (Figure 1B arrow). There was no evidence of inflammatory cells in the alveolar parenchyma and airway spaces, nor were any fibrotic changes observed. Careful analysis of the lungs exposed to doxycycline showed the emergence of clusters of cuboidal cells already after three days of doxycycline treatment (Figure 1C, arrows). Next, we analyzed the extend of the transgene expression after the specified time points by immunohistochemistry with an antibody against the myc epitope (Myc), which is present at the N-terminus of the iSox2 transgenic protein. In contrast to lungs of wild type control or non-induced transgenic mice (Figure 1D), clear positive cells were already detected after one day of doxycycline exposure in subsets of type II cells in the lungs of the iSox2^SPC-rtTA^ mice (Figure 1E), which progressively increased with prolonged exposure to doxycycline for 3, 6, 9 and 28 days (Figure 1F–I). Moreover, the iSox2 expressing cells were positive for the type II cell marker Prospc, indicating that indeed the transgene was expressed in type II cells (Figure 1J–L, arrows indicate positive clusters; Figure S1). Although the appearance of positive cells after one day did not change the overall structure of the lung, the gradual increase of Sox2 positive cells caused cellular changes, from typical type II cells to cuboidal shaped cells. The appearance of these clusters of cuboidal cells suggested that the transgenic iSox2 induced cellular changes in the type II cells. Therefore, we analyzed the expression of the type II cell differentiation marker lysophosphatidylcholine acyltransferase 1, Lpcat1, which is involved in the production of the main phospholipid of surfactant, dipalmitoylphosphatidylcholine. Lungs of iSox2^SPC-rtTA^ were analyzed after the induction of the transgene with dual immunofluorescence staining (Figure 2). Colocalization of Lpcat and iSox2 was observed after one (Figure 2A) and six (Figure 2B) days of doxycycline, but vanished after longer exposure with doxycycline (Figure 2C), indicating that these cells lost the differentiated type II cell characteristics. Thus, expression of iSox2 in peripheral respiratory epithelial cells expanded with the duration of doxycycline administration and induced the appearance of clusters of cuboidal shaped cells, leading to disorganized alveolar septa and loss of the normal lung architecture.

### Sox2 induces proliferation in alveolar type II cells *in vivo*

**Figure 3:**
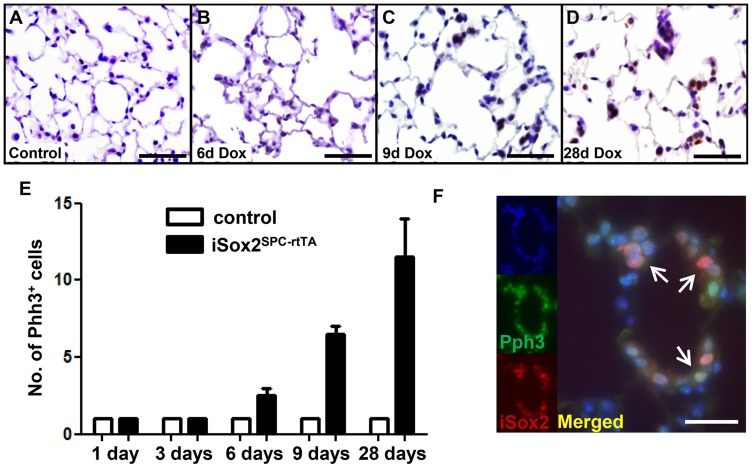
Sox2 induces proliferation in terminally differentiated alveolar type II pneumocytes. Lungs of control (A) and iSox2^SPC-rtTA^ mice treated with doxycycline for 6 (B), 9 (C) or 28 (D) days were analyzed with an antibody against the mitotic cell marker Phh3. Representative images show proliferation in individual type II cells after 6 days of iSox2 induction (B), which gradually develop into proliferative clusters of cells (C, D). (E) Quantification of Phh3 staining indicates the correlation between the increase of Phh3 positive cells and time of doxycycline expression. (F) Colocalization of iSox2 and Phh3 is shown by dual immunofluorescence labeling after 28 days of doxycycline exposure (areas arrows indicate double positive cells). Scale bars: 200 µm (A), 100 µm (B, C, D) and 25 µm (F).

Although cell turnover and proliferation is low during homeostasis in mature lungs, we and others previously showed that Sox2 induced proliferation in lung epithelium [18], [19], [46]. Since Sox2 induced the appearance of an increasing number of cell clusters within the alveolar epithelium, we analyzed whether Sox2 induced proliferation in fully differentiated alveolar type II cells using an antibody against the mitotic cell marker phospho-histone H3 (Phh3). Contrasting the non-proliferative, homeostatic lungs derived from control lungs, Phh3 positive cells were observed in the cuboidal clusters of the lungs of iSox2^SPC-rtTA^ exposed to doxycycline (Figure 3A versus 3B–D). The number of proliferative cells gradually increased with the duration of transgene expression in iSox2^SPC-rtTA^, starting to become apparent after six days of induction (Figure 3E). Dual immunofluorescence staining revealed co-localization of Phh3 and iSox2 in the induced clusters of alveolar cells, confirming that proliferation occurred within the transgene-expressing cells (Figure 3F; Figure S2). So, iSox2 induced type II cells to proliferate and induced cellular changes, ultimately leading to abnormal lung architecture.

### iSox2 induces expression of Clara-like and basal-like cells in AVTII cells

**Figure 4:**
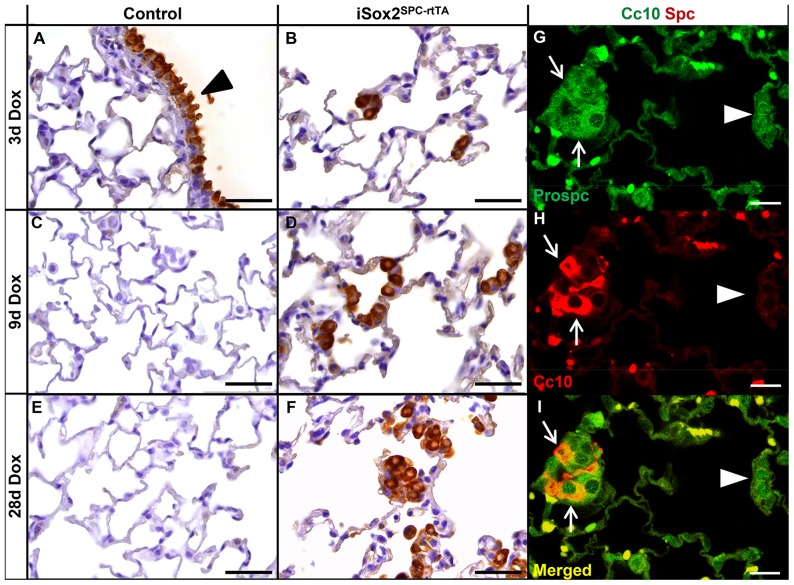
Sox2 induces Clara-like cells and BASC cells. Lungs of control (A, C, E) and iSox2^SPC-rtTA^ (B, D, F) lungs treated with doxycycline for 3 (A, B), 9 (C, D) and 28 (E, F) days were stained with the Clara cell marker Cc10. Endogenous expression of Cc10 is demonstrated in the conducting airways (A, arrowhead), which also shows the absence of Cc10 positive cells in the distal airways (A, C, E) of control lungs. The progressive increase in number of Clara-like cells (Cc10^+^) in the iSox2^SPC-rtTA^ lungs with prolonged induction of iSox2 is clearly noticeable (B, D, F). Colocalization of Cc10 (red) and Prospc (green) is demonstrated with dual immunofluorescence staining (G–I), indicating the emergence of BASC cells (arrows). Arrowheads show Prospc positive cells that lack CC10 (and iSox2) expression. Scale bars: 50 µm (B, D, F), 25 µm (G–I).

Since iSox2 induced the emergence of proximal cell types when expressed during lung development [18], [46], we analyzed if the expression of iSox2 in terminal differentiated type II cells also induced genes specific for proximal airway cells differentiation. Therefore, the expression of Cc10 and Trp63, two markers of the proximal Clara cells and basal cells, respectively, was analyzed. Although Cc10 positive cells were present in the proximal epithelium of the control lungs, the alveolar regions were completely devoid of them (Figure 4A, C, E). In contrast, Cc10 positive cells were readily detected in the alveolar regions in the lungs of the iSox2^SPC-rtTA^ mice, even after one day of doxycycline induction, and increased with prolonged exposure (Figure 4B, D, F). Interestingly, these CC10 positive cells also expressed the type II cell marker Spc, indicating that the iSox2 transgene induced the emergence of a transient, bronchioalveolar stem cell (BASC)-like population (CC10^+^/Spc^+^; arrows in Figure 4G–I; arrowheads indicate CC10 negative cells). The BASC population has been described to serve as a progenitor like population which is induced upon damage to repopulate the airway epithelium [49], [50].

### Ectopic Sox2 expression induces progenitors-like cells

**Figure 5:**
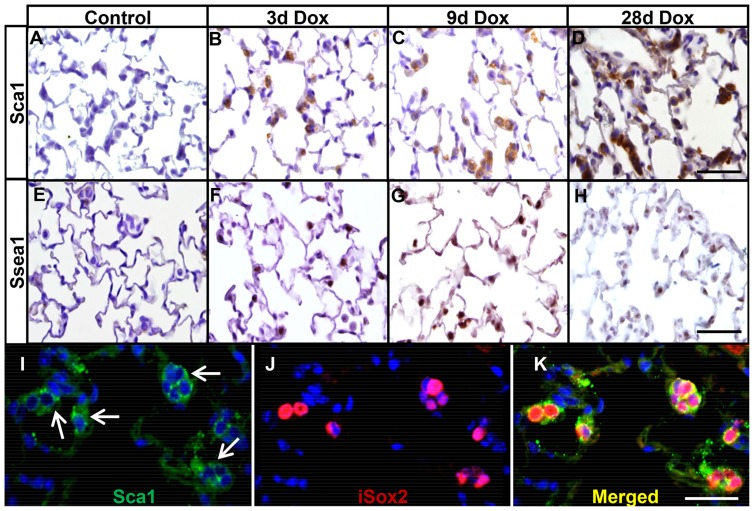
iSox2 induces the appearance of stem cell markers. Immunohistochemistry for Sca1 (A–D) and Ssea1 (E–H) was performed on lungs of controls (A, E), and of iSox2^SPC-rtTA^ mice after 3 (B, F), 9 (C, G) and 28 (D, H) days of doxycycline exposure. Sca1 and Ssea1 expressing cells are completely absent in control lungs (A, E), but readily detectable after 3 days of exposure and progressively increased with duration of transgene activation. Sca1 is clearly associated with iSox2^+^ cells, as shown by dual immunofluorescence staining (I–K). Scale bar 100 µm (D, H), 50 µm (K).

**Figure 6:**
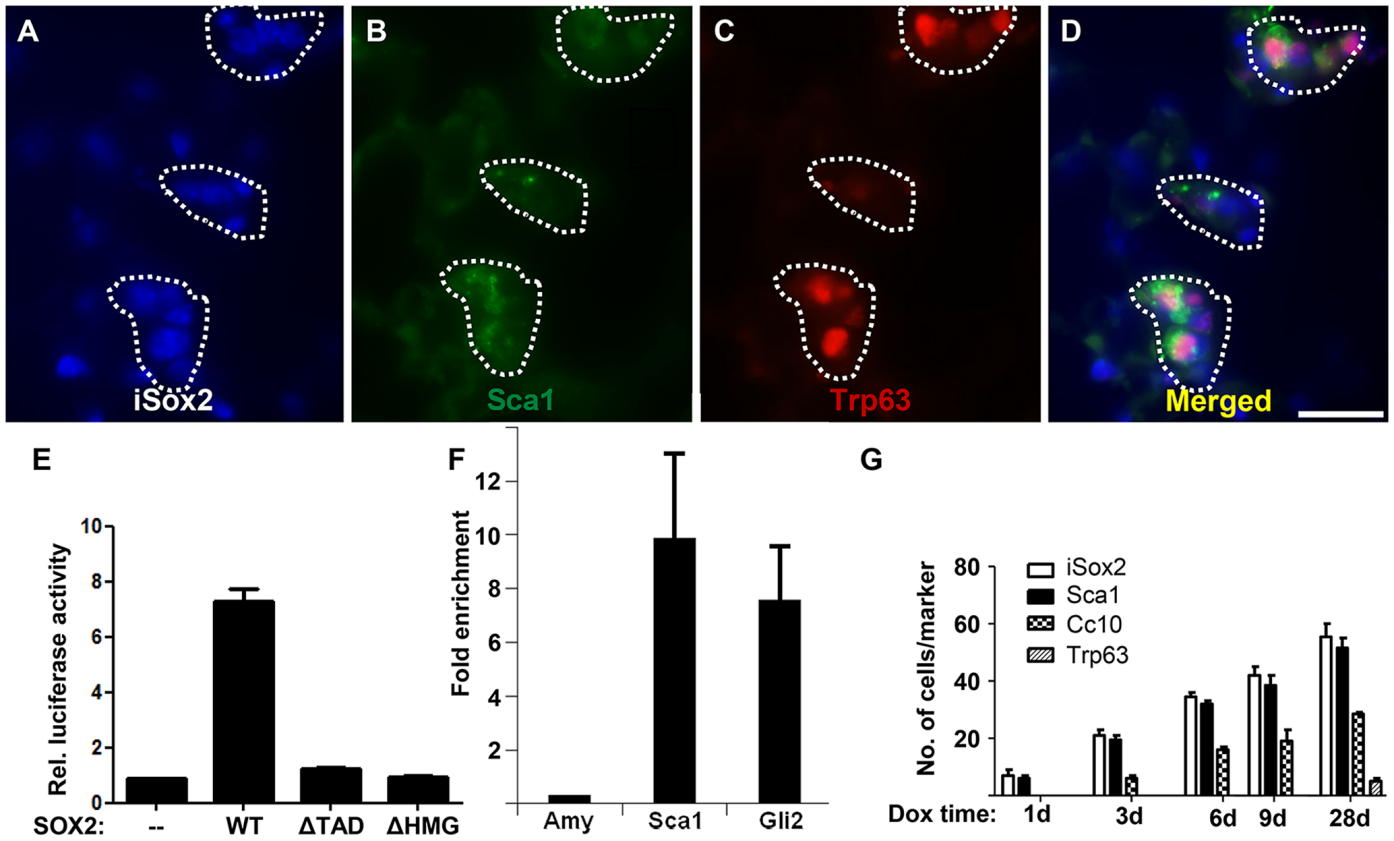
Sox2 induces Sca1 positive cells by directly transactivating the Sca-1 gene. Triple immunofluorescence staining with Myc (iSox2, blue), Sca1 (green) and Trp63 (red) on lungs of iSox2^SPC-rtTA^ animals treated for 28 days with doxycycline demonstrate the emergence of Sca1/Trp63 positive cells (dotted areas; A–D). (E) Luciferase assay shows in vitro transactivation of the Sca1 minimal promoter by the full length Sox2 (WT), but not by deletion constructs lacking the transactivation domain (ΔTAD) or the HMG domain (ΔHMG). The graph represents the average of three independent experiments, and the bars denote the standard error of the mean (SEM). (F) SOX2 specific ChIP analysis showing specific enrichment of the Sca1 promoter region used in the luciferase assay. The SEM is indicated of two independent experiments. (G) Quantification of the expression of the different lung markers in vivo after the indicated time of doxycycline exposure. The graph shows the average of the number of positive cells of five microscopic fields with a 40× objective of three independent experiments, including the corresponding SEM. Scale bar: 25 µm (D).

Since we observed the emergence of proximal markers in the distal type II cells and the appearance of Spc^+^/CC10^+^ double positive BASC-like cells, we wondered whether the differentiation of proximal Clara and basal cells occurred through intermediate, progenitor-like cells. Therefore, we analyzed the lungs of control and iSox2^SPC-rtTA^ mice, isolated at different time points after doxycycline treatment, for the expression of Sca1 and Ssea1, two markers normally expressed in progenitor cells [51], [52]. Sca1and Ssea1 were readily detected in the alveolar epithelium in iSox2^SPC-rtTA^ after 3 days of doxycycline exposure to these mice (Figure 5B, F). This pattern of expression progressively increased after 9 days of transgene induction (Figure 5C, G), and after four weeks of doxycycline induction virtually all clusters of cells expressed Sca1 and Ssea1 (Figure 5D, H). Sca1 and myc-epitope double immunofluorescence staining on lungs of iSox2^SPC-rtTA^ animals exposed for four weeks to doxycycline clearly showed co-localization of Sca1 with the transgenic protein (Figure 5I–K). Moreover, the previously identified Spc^+^/CC10^+^ cells also appeared to express Sca1. In addition, Sca1 also co-expressed with the basal cell marker Trp63 after four weeks of iSox2 induction, suggesting that the Sca-1^+^ cells gradually differentiate into more committed cells (Figure 6A–D; Figure S3). Moreover, the Trp63 positive cells represent the ectopic appearance of basal-like cells in the distal epithelium of the iSox2^SPC-rtTA^ lungs. Thus, Sca1 was specifically induced in the subset of AVTII cells that expressed transgenic iSox2, which indicates that iSox2 is able to induce progenitor-like cells in terminally differentiated type II cells.

### Sox2 activates promoter-Luciferase construct for Sca-1

Next, we wondered whether Sca1 expression is directly regulated by Sox2. Therefore, the Sca1 gene was analyzed for putative Sox2 binding sites and within a region of 500 bp immediately upstream of the transcriptional start site, a well-conserved Sox2 binding motif was found. The functionality of this potential Sox2 site was tested in vitro using a luciferase reporter assay. The full length Sox2 protein (WT) induced the transcriptional activity of the Sca1-luciferase construct sevenfold as compared to baseline expression, whereas mutant Sox2 proteins lacking either the transactivation domain (ΔTAD) or the HMG domain (ΔHMG) did not transactivate the minimal Sca1 promoter (Figure 6E). Next, the in vivo binding of SOX2 to this putative Sox2 binding site was analyzed by chromatin immunoprecipitation (ChIP), which revealed that SOX2 directly bound to this Sox2 motif of the SCA1 gene in the human bronchiolar cell line A549 (Figure 6F). These results demonstrated the direct binding and activation of the Sca1 promoter by Sox2, and thus highlights a novel transcriptional target gene of Sox2.

### Transgenic Sox2 induces proximal markers in primary AVTII cells

**Figure 7:**
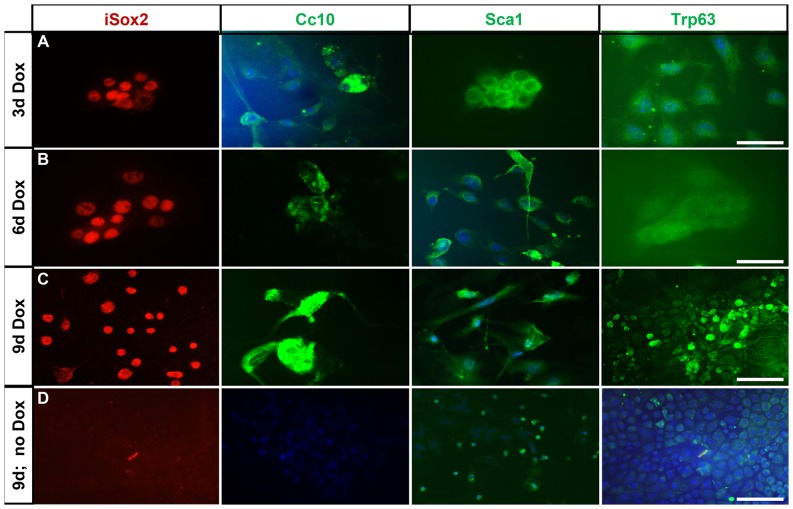
Primary alveolar type II cells can be reprogrammed *in vitro*. Representative immunofluorescence images showing expression of iSox2 (Red), Cc10 (Green), Sca1(Green) and Trp63 (Green) after culturing the primary type II cells with doxycycline for 3 days (A), 6 days (B) and 9 days (C). AVTII cells cultured for 9 days without doxycycline served as negative controls (D). The results demonstrate that in vitro cultures of AVTII cells have comparable phenotypic plasticity as AVTII cells in vivo. Scale bar 50 µm (A–C), 100 µm (D).

Quantification of the appearance of the different markers and cell types in relation to the timing of doxycycline exposure, suggested that Sox2 first initiates the appearance of markers associated with progenitor-like cells (Figure 6G; Sca1^+^). In time, differentiation markers emerge, as evidenced by the number of Cc10^+^ and Trp63^+^ cells. To determine the observed plasticity of AVTII cells in vitro, we isolated type II cells from 4 weeks old non-doxycycline exposed iSox2^SPC-rtTA^ mice and cultured these primary cells with or without doxycycline (Figure S4). After three, six and nine days the cultures were analyzed for the expression of the myc epitope (iSox2), Cc10, Sca1 and Trp63. The non-doxycycline treated cells expressed Spc, indicative for AVTII cells, but obviously lacked expression of Cc10, Sca1 and Trp63 (Figure 7D). However, in cells treated with doxycycline for 3 days (Figure 7A), 6 days (Figure 7B) and 9 days (Figure 7C), AVTII cells expressed Sca1, and Cc10, while Trp63 positive cells were observed only in the cultures exposed to doxycycline for nine days, which correlated with the in vivo observations (Figure 6D). Taken together, these data demonstrate that AVTII cells also exhibit phenotypic plasticity in vitro.

## Discussion

The idea that the future potential of differentiated cells is limited has been gradually adapted, starting with the seminal work of nuclear replacement in frogs and culminated in the complete reversal of differentiated fibroblast into multipotent cells by only four factors [23], [53]. These experiments have led to an expansion of experimental approaches to manipulate and modify cells to give them multipotent potential [54]. Several adaptations to this original combination of four factors have been shown to result in pluripotent cells, depending on the starting source of cells used as target [55]–[58]. Recently, in vivo reprogramming has been show, for instance of resident brain astrocytes in both young adults and mice with Sox2 only [31].

Our previous study established that ectopic expression of Sox2 during lung development caused cystic lesions and aberrant differentiation of the epithelial cells [18]. Recently, we showed that Sox2 directly induced Trp63 and Gata6 expression, which caused naïve epithelial cells to be become unresponsive to the branch inducing signal Fgf10 [46]. This led to a skewing of the developmental potential of the uncommitted cells towards a proximal cell fate, primarily basal cells. In the current study we investigated whether Sox2 would be sufficient to redirect the fate of terminally differentiated alveolar type II cells.

After bleomycin induced injury, type II cells start to proliferate and repopulate the alveolar epithelium by self-renewal and functioning as progenitor cells for type I cells [59]. We showed that induction of Sox2 expression in alveolar type II cells resulted in the immediate emergence of proliferative cells. Sox2, together with the Wnt downstream mediator β-catenin, was shown to directly regulate the promoter of Ccnd1 in breast cancer cells [60], and we show that the iSox2 positive cells were expressing Pph3 and cyclinD1. Sox17 was also suggested to regulate the Ccnd1 promoter directly, although no ChIP analysis was done [61]. Tompkins et al. added that upon Sox2 expression several other genes involved in cell cycle initiation and progression were upregulated [19]. Within the first week of doxycycline induction, the iSox2 expressing cells started to express the Sca1 and Ssea1 stem cell markers [51], [52]. Moreover, we showed a direct binding and transactivation of the Sca1 promoter by Sox2, thereby initiating a progenitor-like program. This suggests that iSox2^+^/Sca1^+^/Ssea1^+^ cells represent an initial sign of dedifferentiating type II cells, indicating that Sox2 may initiate alveolar epithelial cell plasticity by first regulating the emergence of proliferative intermediate cells, perhaps progenitor-like cells. Thus, our data suggest a mechanism where Sox2 first induces proliferative Ssea1^+^/Sca1^+^ progenitor cells, which increase over time and subsequently promote differentiation of these cells into proximal epithelial cells. Detailed analysis of the progenitor-like, iSox2/Sca1^+^/Ssea1^+^ cells showed a gradual differentiation towards proximal epithelial cell fate, since these cells started to express Cc10 and Trp63 after longer exposure to doxycycline, both in vivo and in vitro. A number of these cells co-expressed Spc and Cc10, a population also referred to as the bronchioalveolar stem cells (BASC), which are normally located at the bronchio-alveolar junctions [49]. These authors also showed that the BASC cells expressed Sca1, which became proliferative after naphthalene injury. The cells with characteristics of BASC cells in the iSox2 expressing lungs also expressed Sca1 (Spc^+^/Cc10^+^/Sca1^+^), contrasting earlier findings obtained with ectopic Sox17 expression that did not find these triple positive cells [61]. The Sca1 marker has been used to purify BASC through fluorescence activated cell sorting (FACS) using CD45^−^CD31^−^Sca1^+^ [49], [62], but other studies described this population to be more heterogeneous [63]. BASC cells self-renew and have the potential to differentiate into proximal and distal epithelial cells [34]. Lineage tracing experiments using a CCSP-CreEr followed by hyperoxia injury suggested that BASC cells did not give rise to alveolar cells [50], but after bleomycin injury they did [59]. This would suggest that BASC cells respond differently to various triggers. Recently, lineage tracing studies using bleomycin induced lung damage in Scgb1a1-CreER mice showed that basal cells (Trp63^+^) in the damaged parenchyma were directly derived from Clara cells (Scgb1a1^+^) [64]. Moreover, upon SO_2_-induced damage or viral infection, Clara cells also dedifferentiated into basal cells [65]. Exposure to naphtalene or hyperoxia revealed that Clara cells may contribute to maintenance and repair of the conducting airways without dedifferentiating into basal cells [50]. Our current findings demonstrate that Clara and basal cells may originate from iSox2^+^/Sca1^+^ progenitor cells. However, it remains to be determined whether these cells are derived from Sca1^+^ progenitor cells by genetic lineage tracing experiments.

The continuous proliferation of iSox2 positive cells led to the emergence of clusters of cells in the alveolar walls with a cuboidal to columnar appearance, which became apparent after 9 days. Concomitant with the development of these clusters was the disruption of the alveolar structure, as evidenced by the emphysema-like phenotype observed after four weeks of iSox2 expression. The combination of proliferation and the induction of progenitor-like characteristics resulted in the loss of structural integrity. The conversion of cell fate combined with the increased proliferation induced by iSox2 may have changed the secretion and composition of the extra cellular matrix, which may have weakened the alveolar structure. This in turn may enhance the activity of local proteases to digest the tissue matrix and induce septal rupture, leading to emphysematous-like lungs.

Long term and high ectopic expression of Sox2 in Cc10 positive cells was shown to result in adenocarcinomas in fifty percent of the mice [66], but we and others did not find evidence that Sox2 induced lung cancer in our mouse models [19]. The difference in the various transgenic approaches may contribute to this discrepancy. However, SOX2 has been associated with human squamous cell lung tumors (Bass et al, 2009; Hussenet et al, 2010; Yuan et al, 2010).

The only solution for patients with end-stage, severe chronic lung disease, like COPD and idiopathic pulmonary fibrosis, is lung transplantation. However, the shortage of suitable donors may result in a significant mortality of patients. Therefore, a potential future treatment for these severe lung diseases is a (temporary) transplantation with engineered lungs or stem/progenitor cells [67]–[69]. However, the approaches for generating these cells have been limited to the use of combination of factors in vitro [23], [34], [70], [71]. In addition, a variety of in vitro protocols exist for differentiating a range of pulmonary epithelial cell types, including alveolar type II cells [67]–[69], [72]–[74]. Recently, the direct conversion of cellular fate has been reported in vivo in a study demonstrating that neurons can be generated from endogenous mouse astrocytes that are reprogrammed by viral delivery in situ [75] In addition, it has been shown that SOX2 is also capable of converting resident astrocytes into proliferative neuroblasts [31]. We showed that Sox2 alone is sufficient to induce alveolar plasticity in resident lung alveolar type II cells into progenitors in adult mice. Our study demonstrates a feasible strategy for using Sox2 to reprogram alveolar type II cells in vivo and in vitro. In the future, studies to identify the signaling pathways that regulate the differentiation of progenitors and the induction of proliferation in alveolar type II cells will be critical to facilitate the understanding of alveolar plasticity for future regenerative medicine. Lineage reprogramming would be applicable in translational medicine if this event can be triggered by a factor, whether transcription factor or small molecule, which acts transiently and exerts a complete effect.

In conclusion, we ectopically expressed one of the Yamanaka reprogramming factors, Sox2, in type II cells cells using our previously described system [18], [46]. We show that these dedifferentiate into progenitor-like cells and subsequently commit to the proximal pulmonary epithelial cell lineages, like basal cells, extending previous findings with Sox2 and Sox17 [19], [61]. Moreover, we show that aside from directly activating the promoter of the key gene in basal cell development, Trp63 [46], Sox2 also binds and activates the progenitor cell marker Sca1, providing molecular evidence for a direct role of Sox2 in the dedifferentiation process.

## Supporting Information

Figure S1 Dual immunofluorescence staining shows the colocalization of the transgenic Sox2 (iSox2, red) with the type II cell marker Prospc (green) after 6 days (A), 9 days (B) and 28 days (C) of doxycycline treatment. Scale bars 25 µm.
(TIF)

Figure S2 Colocalization of iSox2 and Phh3 is shown by dual immunofluorescence labeling after 28 days of doxycycline exposure. Scale bars 25 µm.
(TIF)

Figure S3 Triple immunofluorescence staining with Myc (iSox2, blue), Sca1 (green) and Trp63 (red) on lungs of control (A–D) and iSox2^SPC-rtTA^ (E–H) animals treated for 28 days with doxycycline demonstrate the emergence of Sca1/Trp63 positive cells (dotted areas; E–H).
(TIF)

Figure S4 Immunofluorescence staining with Prospc (A) or Lpcat1 (B) of isolated type II cells after one day in culture, showing a high percentage of positive cells after the isolation.
(TIF)
