# Supplementary figures and images for: Differentiated Type II Pneumocytes Can Be Reprogrammed by Ectopic Sox2 Expression
Source: PLoS One. 2014 Sep 11;9(9):e107248. doi: 10.1371/journal.pone.0107248 (PMC4161395; doi:10.1371/journal.pone.0107248)

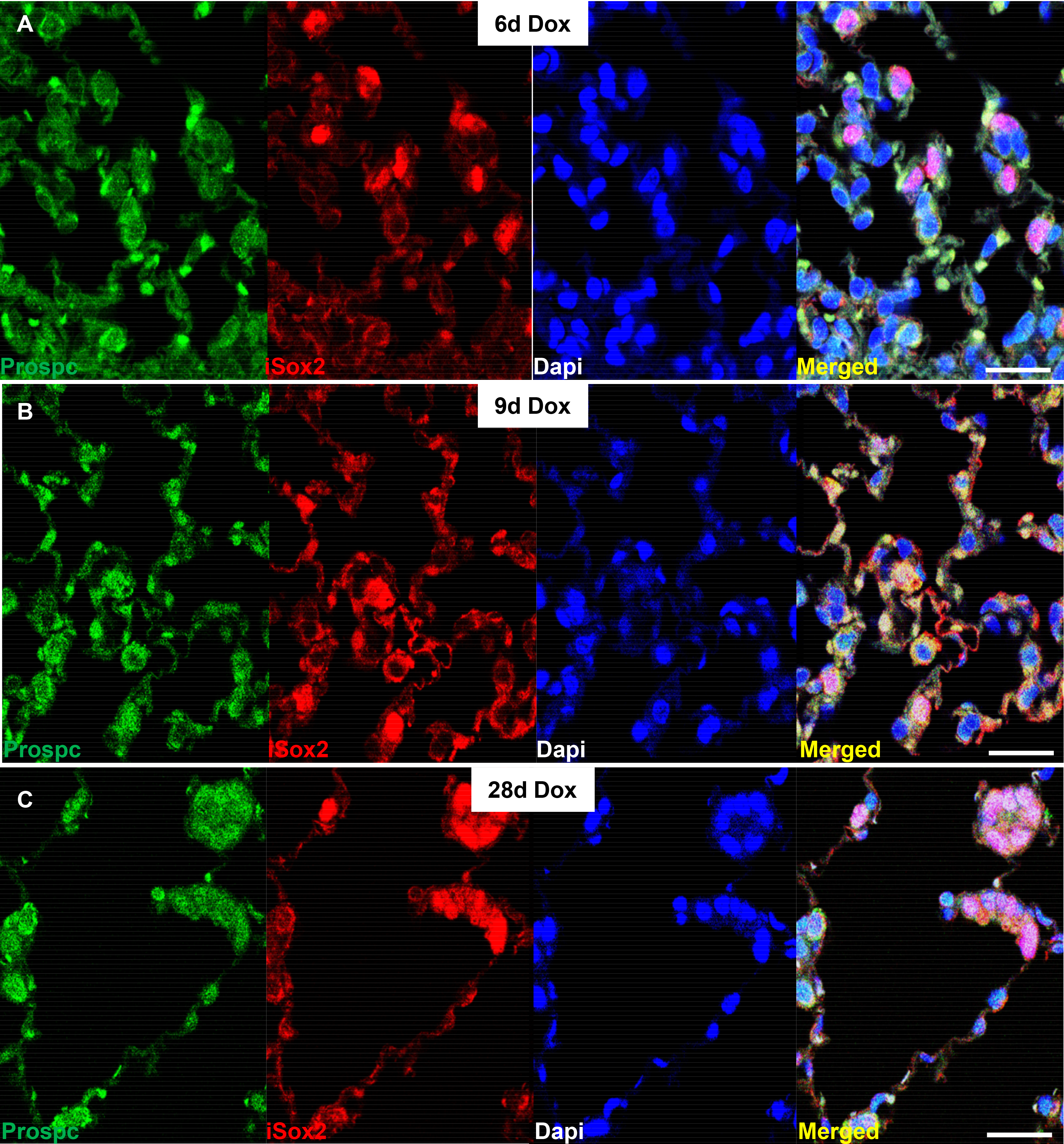

Supplement: Figure S1 — Dual immunofluorescence staining shows the colocalization of the transgenic Sox2 (iSox2, red) with the type II cell marker Prospc (green) after 6 days (A), 9 days (B) and 28 days (C) of doxycycline treatment. Scale bars 25 µm. (TIF) [file pone.0107248.s001.tif]

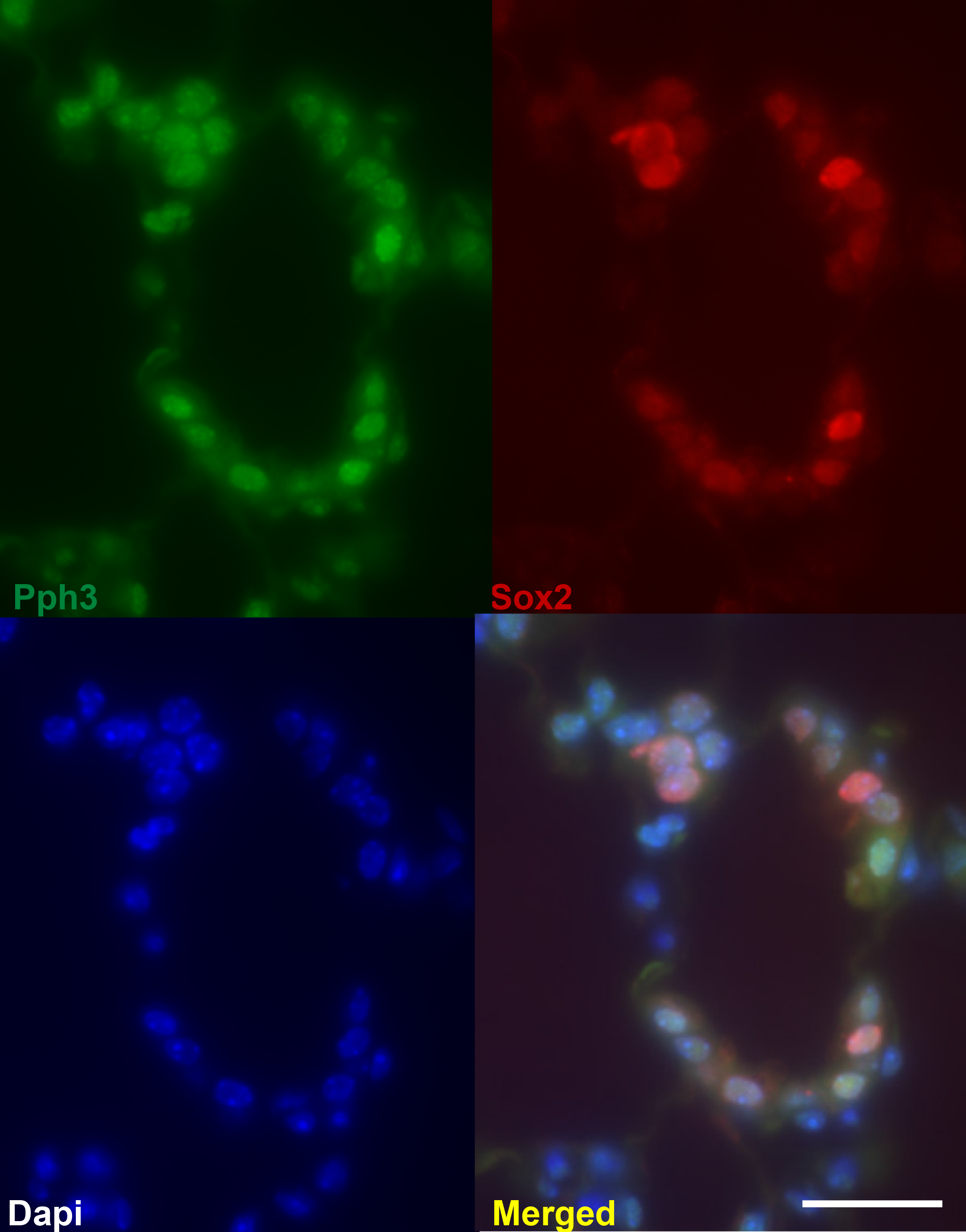

Supplement: Figure S2 — Colocalization of iSox2 and Phh3 is shown by dual immunofluorescence labeling after 28 days of doxycycline exposure. Scale bars 25 µm. (TIF) [file pone.0107248.s002.tif]

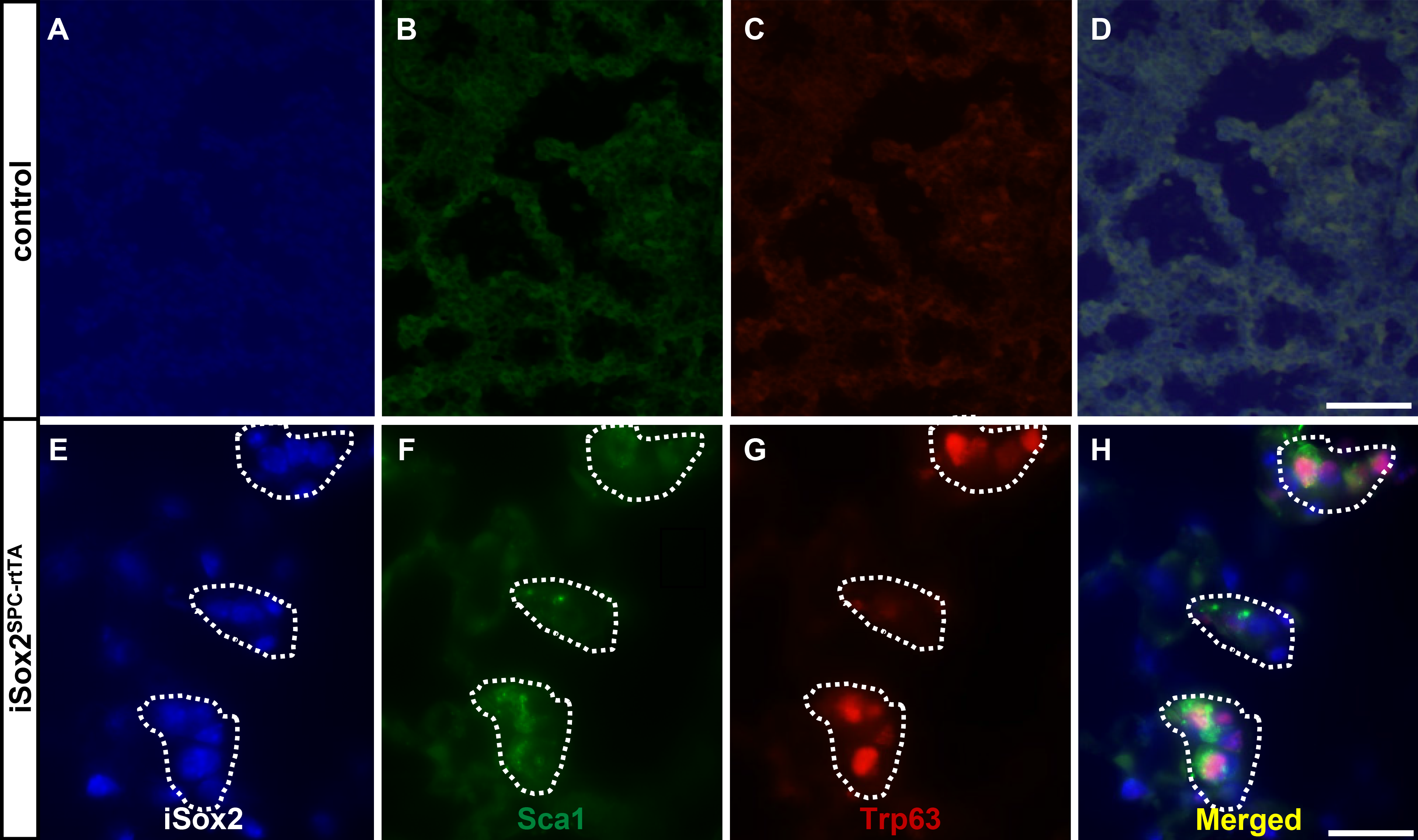

Supplement: Figure S3 — Triple immunofluorescence staining with Myc (iSox2, blue), Sca1 (green) and Trp63 (red) on lungs of control (A–D) and iSox2SPC-rtTA (E–H) animals treated for 28 days with doxycycline demonstrate the emergence of Sca1/Trp63 positive cells (dotted areas; E–H). (TIF) [file pone.0107248.s003.tif]

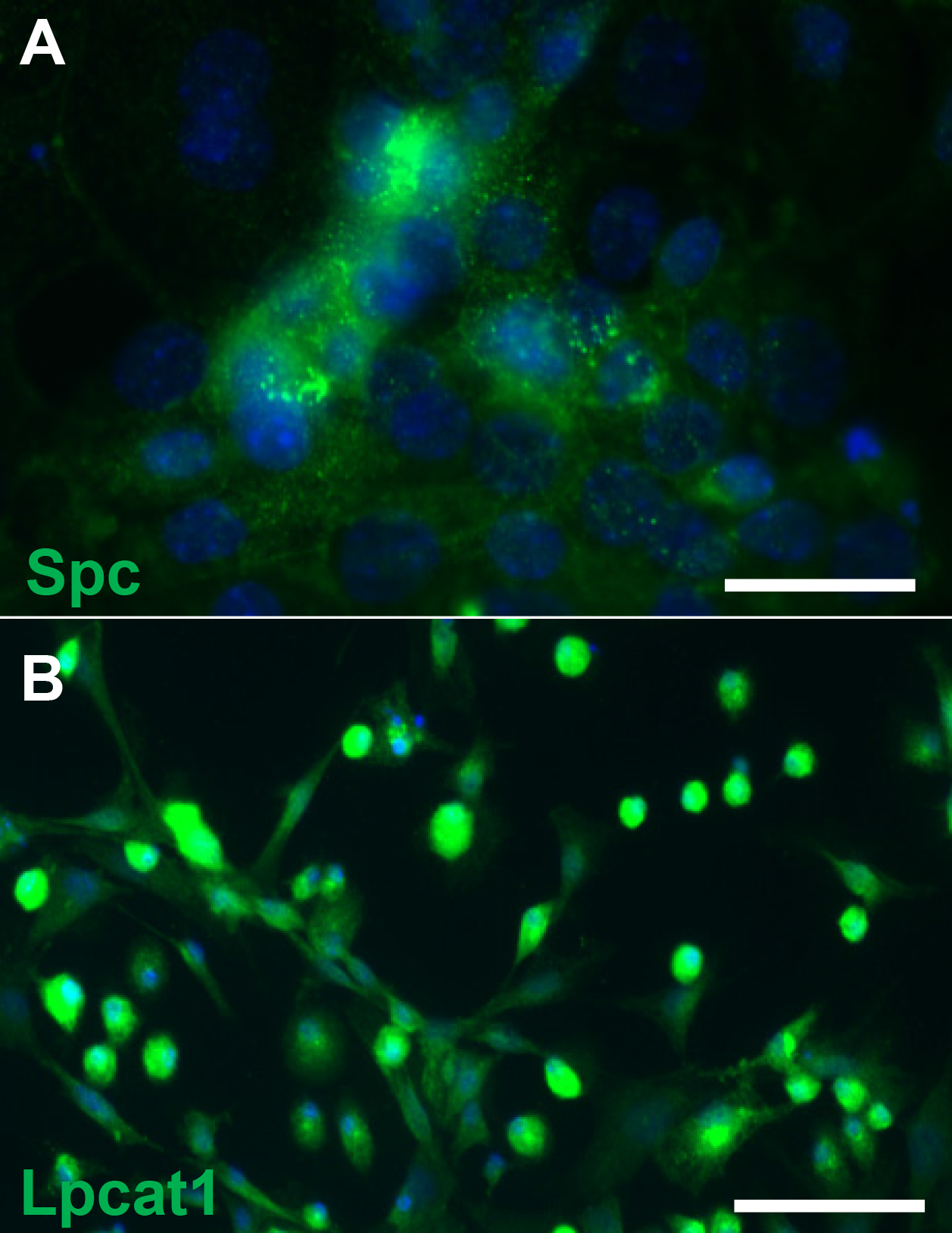

Supplement: Figure S4 — Immunofluorescence staining with Prospc (A) or Lpcat1 (B) of isolated type II cells after one day in culture, showing a high percentage of positive cells after the isolation. (TIF) [file pone.0107248.s004.tif]
